# Predictors of discontinuation of osteoporosis treatment: sub-analysis of the Japanese osteoporosis intervention trial-05 (JOINT-05)

**DOI:** 10.1007/s00774-024-01541-3

**Published:** 2024-08-14

**Authors:** Yasuhiro Takeuchi, Yuki Nakatsuka, Shiro Tanaka, Tatsuhiko Kuroda, Hiroshi Hagino, Satoshi Mori, Satoshi Soen

**Affiliations:** 1 Toranomon Hospital Endocrine Center, 2-2-2 Toranomon, Minato-ku, Tokyo, 105-8470 Japan; 2 Okinaka Memorial Institute for Medical Research, Minato-ku, Tokyo, Japan; 3 Department of Clinical Biostatistics, Graduate School of Medicine, Kyoto University, Kyoto, Japan; 4 Public Health Research Foundation, Shinjuku-ku, Tokyo, Japan; 5 Department of Rehabilitation, Sanin Rosai Hospital, Yonago, Tottori Japan; 6 Seirei Hamamatsu General Hospital, Hamamatsu, Shizuoka, Japan; 7 Soen Orthopaedics, Osteoporosis and Rheumatology Clinic, Kobe, Hyogo Japan

**Keywords:** Treatment discontinuation, Predictor, Teriparatide, Alendronate

## Abstract

**Introduction:**

To identify predictors of discontinuing treatment with teriparatide (TPTD) and alendronate (ALN), data from a randomized, controlled trial (JOINT-05) involving postmenopausal Japanese women at high risk of fracture were re-analyzed.

**Materials and Methods:**

Participants received sequential therapy with once-weekly TPTD for 72 weeks followed by ALN for 48 weeks (TPTD-ALN group) or monotherapy with ALN for 120 weeks (ALN group). Background data including comorbidities, fracture prevalence, cognitive function, quality of life, activities of daily living, bone metabolism parameters, and nutrient intake were collected. The endpoints were 3 types of discontinuations by the reason: a poor compliance, adverse events (AEs), or any reason including those unrelated to AEs or poor compliance. Odds ratios (ORs) of baseline predictors of discontinuation were evaluated by single or multiple regression analysis.

**Results:**

A total of 234 (49.0%) patients in the TPTD-ALN group and 167 (34.2%) patients in the ALN group discontinued. In the TPTD-ALN group, a lower serum calcium level was a significant predictor of compliance-related discontinuation. Serum 25-hydroxyvitamin D levels were lower in patients with lower serum calcium levels than with higher serum calcium levels. In the ALN group, poor cognitive function was significantly associated with compliance-related discontinuation, and higher body mass index and alcohol intake were predictors of AE-related discontinuation. Predictors of discontinuation were drug-specific. Lower serum calcium levels and poor cognitive function were predictors of discontinuing once-weekly TPTD and ALN, respectively.

**Conclusion:**

When starting TPTD and ALN treatment, careful attention to patients with lower serum calcium levels and poor cognitive function, respectively, may be needed for better treatment continuity.

**Supplementary Information:**

The online version contains supplementary material available at 10.1007/s00774-024-01541-3.

## Introduction

Osteoporosis is a common chronic skeletal disease characterized by decreased bone mass, loss of skeletal integrity, and increased susceptibility to fragility fractures [1]. Fragility fractures are associated with hospitalization, mortality, and increased healthcare costs [2]. Osteoporosis is treated with bone resorption inhibitors or bone anabolic agents, and the most common challenge is maintaining patients’ continuation to treatment. As in many chronic diseases, discontinuation of treatment by poor adherence imposes a significant burden on both patients and healthcare systems [3]. In fact, it has been reported that poor compliance, defined as a medication possession ratio less than 80%, was associated with a 17% increase in the fracture rate, a 37% increase in the risk of all-cause hospitalization, and increased costs [4].

Several strategies have been used to prevent discontinuation of osteoporosis treatment. A difference in the dosing regimen of bisphosphonates affects adherence, resulting in certain improvements [5]. It has been reported that systems that support patient decision-making may also be useful in preventing dropouts [6].

In addition, increasing refill compliance levels was found to be associated with progressively lower fracture rates [7]. Improved osteoporosis medication adherence can also reduce osteoporosis-related healthcare costs by preventing fractures [8]. Moreover, all-cause mortality rates were lower in patients with good adherence to anti-osteoporosis medications [9].

To improve adherence to drugs for osteoporosis, several studies have reported predictors of poor adherence. Factors such as patients’ health attitudes, inadequate patient education, and aging act as barriers to adherence [10].

For once-weekly teriparatide (TPTD), older age, starting administration while hospitalized, and side effects have been identified as factors decreasing continuation [11]. However, no reports have comprehensively evaluated the relationships between discontinuation and the subject’s background characteristics, including comorbidities, prevalence of fracture, cognitive function, quality of life (QOL), activities of daily living (ADL), and laboratory measurements. Furthermore, there have been no reports comparing discontinuation to different types of drugs in randomized, controlled trials involving patients with osteoporosis.

To identify the predictors of discontinuation of once-weekly TPTD and alendronate (ALN), data from a randomized, controlled trial (JOINT-05) involving postmenopausal Japanese women at high risk of fracture, in which comprehensive data collection was conducted, were re-analyzed [12].

## Materials and methods

### Participants and treatment

The JOINT-05 trial was a prospective, randomized, open-label, blinded-endpoint, pragmatic, effectiveness trial performed in Japan [13]. Japanese women at least 75 years of age with primary osteoporosis [14] were randomly allocated to two groups: sequential therapy with once-weekly TPTD for 72 weeks followed by once-weekly ALN for 48 weeks (TPTD-ALN group); or monotherapy with ALN for 120 weeks (ALN group). Native vitamin D (400 IU/day) was provided to both groups throughout the entire treatment period. Patients at high risk of fracture, defined by lower bone mineral density (BMD) and higher number or grade of prevalent vertebral fractures, were included. The detailed inclusion criteria have been described elsewhere [13]. The protocol was conducted according to the Declaration of Helsinki. Informed consent was obtained from all individual participants included in the study. This study was approved by the central and onsite institutional review boards (IRBs). For this sub-analysis, a dataset of participants enrolled in JOINT-05 [12] that excluded the data of those with hypercalcemia at baseline was analyzed.

### Baseline measurements

Age, age at menopause, years from menopause, and history of prior treatment were investigated. Numbers and grades of prevalent vertebral fractures were evaluated by the semi-quantitative method [15]. BMD was measured at the lumbar spine, total hip, and femoral neck in each institution by dual-energy X-ray absorptiometry. As comorbidities, the prevalences of hypertension, diabetes mellitus, dyslipidemia, and rheumatoid arthritis were investigated. Each participant’s cognitive level was evaluated by the Mini-Mental State Examination (MMSE) [16].

Physical function was evaluated with the timed-up-and-go test (TUG) and the one-leg standing test with eyes open (OLST). Nursing level support required was also investigated. Body weight and height were measured, and the body mass index (BMI) was calculated using the formula: body weight (kg) divided by the square of height (m^2^).

Urinary pentosidine levels and serum levels of osteocalcin, procollagen type 1 N-terminal propeptide (P1NP), tartrate-resistant acid phosphatase 5b (TRACP-5b), 25-hydroxyvitamin D (25OHVD), hemoglobin A1c (HbA1c), total cholesterol, high-density lipoprotein (HDL) cholesterol, low-density lipoprotein (LDL) cholesterol, triglycerides, creatinine, albumin, and calcium were measured. The estimated glomerular filtration rate (eGFR) was calculated using the following formula: 194 × serum creatinine -1.094 × age^−0.287^ × 0.739.

The degree of back pain was assessed using a visual analog scale (VAS), with scores ranging from 0 to 100 points, at rest and in motion. Quality of life was measured using the EQ-5D [17]. The nutrient intake of the subjects was investigated using a Food Frequency Questionnaire [18].

### Outcome measures

The endpoint was treatment discontinuation during the observation period, which was classified into three types by reason: due to poor compliance; due to AEs; and any reason including those unrelated to compliance or AEs.

The primary outcome of the study was treatment discontinuation due to compliance-related reasons until 120 weeks. Secondary outcomes were treatment discontinuation due to AEs until 120 weeks and any treatment discontinuation until 120 weeks. In JOINT-05, criteria for treatment discontinuation were pre-specified in the protocol: (1) if the participant requests discontinuation from the clinical trial; (2) participants’ reasons such as relocation and busy schedule that make it difficult to continue the study; (3) other reasons that make it difficult to continue the study; (4) AEs that make it difficult to continue the study; (5) worsening of symptoms or other conditions that make a change in treatment necessary; (6) deviations from the eligibility criteria or exclusion criteria identified during the treatment period; (7) withdrawal of TPTD for more than eight consecutive weeks (56 days) during the ever 6 months treatment period (< 66.7% of treatment); and (8) total non-compliance to ALN identified in the survey on the use of ALN. Compliance-related treatment discontinuation was defined as treatment discontinuation due to (1), (2), or (3), which were chosen because they confirmed clear patient intent. AE-related treatment discontinuation was defined as treatment discontinuation due to (4) or (5). Treatment discontinuation for any reason including those unrelated to compliance or AEs was defined as treatment discontinuation due to any of (1) to (8).

### Potential predictors of treatment discontinuation

Potential predictors pre-specified in the statistical analysis plan were: allocation group, age, age at menopause, years since menopause, number of prevalent vertebral fractures, grade of prevalent vertebral fractures, history of femoral fractures, history of osteoporosis treatment, history of bisphosphonate use, BMD, comorbidities, height, weight, BMI (< 18.5 kg/m^2^, 18.5 to 24 kg/m^2^, ≥ 25 kg/m^2^), clinical and laboratory examinations, QOL scores, cognitive function (MMSE), physical function (TUG, OLST), nursing care level, food intake frequency, and results of oral cavity assessment.

### Statistical analysis

Numerical data are presented as means ± standard deviations, whereas categorical data are expressed as numbers and proportions (%). Odds ratios (ORs) and 95% confidence intervals (CIs) were calculated to show the associations between predictors and treatment discontinuation.

Predictors were identified in three analysis populations (TPTD-ALN group, ALN group, and the total population) by two methods independently. One method was the least absolute shrinkage and selection operator (LASSO), an estimation method using the L2 penalty, on multivariable logistic regression analysis. The Schwarz Bayesian criterion and cross-validation were used for selecting the LASSO tuning parameters. The other method was univariable logistic regression. The covariate of each univariable model for which the result of the Wald test was significant was selected. The results of both LASSO and univariable regression are reported, and the predictors to be used in the models were examined based on the results and the clinical significance of each variable. The selected predictors were included in the multivariable logistic regression analysis. Missing covariates were handled using multiple imputation.

All data were analyzed with SAS software version 9.4 (SAS Institute, Cary, NC). P values < 0.05 were considered significant.

## Results

After excluding patients with hypercalcemia (11cases in the TPTD-ALN group and 8 cases in the ALN group) at baseline from the full-analysis population of the JOINT-05 study, 478 patients in the TPTD-ALN group and 488 patients in the ALN group were included in this analysis. During the observation period, the number of patients according to each of the discontinuation criteria was as follows: (1) participant requests: 103; (2) relocation or busy schedule: 82; (3) other reasons: 87; (4) AEs: 107; (5) worsening of symptoms or conditions: 4; (6) deviations from eligibility or exclusion criteria: 7; (7) withdrawal of teriparatide: 8; (8) non-compliance to alendronate: 3. Compliance-related discontinuation was observed in 143 (29.9%) patients in the TPTD-ALN group and 129 (26.4%) patients in the ALN group. AE-related discontinuation was seen in 78 (16.3%) in the TPTD-ALN group and 33 (6.8%) in the ALN group. The number who discontinued for any reason was 234 (49.0%) in the TPTD-ALN group and 167 (34.2%) in the ALN group.

Predictors of discontinuation among the baseline measurements collected were identified by the two prespecified methods. Odds ratios and 95%CIs of all potential predictors for discontinuation on univariable logistic regression are shown in Online Resource 1 (Supplemental Tables 1–3). Weight, serum albumin and calcium levels, vitamin B intake, and folic acid intake were significantly related to compliance-related discontinuation in the TPTD-ALN group. Dyslipidemia, MMSE, weight, and osteocalcin were possible predictors of discontinuation in the ALN group. No significant characteristics were identified for AE-related discontinuation in the TPTD-ALN group, and age at menopause, years from menopause, BMI, eGFR, protein intake, and fat intake were significantly associated with AE-related discontinuation in the ALN group. Serum calcium was a candidate factor for discontinuation for any reason in the TPTD-ALN group, and age at menopause, years from menopause, MMSE, osteocalcin, and P1NP were candidate factors in the ALN group.

A final model to identify predictive parameters for the three types of discontinuation on multiple regression analysis is shown in Table 1. In the TPTD-ALN group, the serum calcium level was significantly associated with compliance-related discontinuation (OR: 0.41, 95% CI: 0.21–0.78, p = 0.01) and discontinuation for any reason (OR: 0.44, 95% CI: 0.28–0.69, p < 0.01). In the ALN group, the prevalence of dyslipidemia (OR: 0.40, 95% CI: 0.20–0.79, p = 0.01), MMSE (OR: 0.92, 95% CI: 0.87–0.97, p < 0.01), weight (OR: 0.97, 95% CI: 0.95–1.00, p = 0.04), and osteocalcin (OR: 1.02, 95% CI: 1.00–1.04, p = 0.05) were significantly associated with compliance-related discontinuation. BMI ≥ 25 kg/m^2^ (OR: 3.93, 95%CI: 1.69–9.14, p < 0.01) and alcohol intake (OR: 1.01, 95% CI: 1.00–1.01, p = 0.01) were also significantly associated with AE-related discontinuation, and MMSE (OR: 0.92, 95%CI: 0.87–0.97, p < 0.01) was the only factor associated with discontinuations for any reason.

**Table 1:** Multivariable logistic regression analysis of associations between treatment discontinuation and significant parameters on univariable analysis

Type of discontinuation	Treatment	Item	OR	95% CI		*p*
Compliance-related	TPTD-ALN	P1NP, 1-μg/L increase	1.00	1.00	1.01	0.05
		Calcium, 1-mg/dL increase	0.41	0.21	0.78	0.01
	ALN	Dyslipidemia, yes	0.40	0.20	0.79	0.01
		MMSE, 1-point increase	0.92	0.87	0.97	< 0.01
		Weight, 1-kg increase	0.97	0.95	1.00	0.04
		Osteocalcin, 1-ng/mL increase	1.02	1.00	1.04	< 0.05
AE-related	TPTD-ALN	Carbohydrate intake, 1-g increase	1.00	0.99	1.00	0.10
	ALN	BMI, ≥ 25 kg/m^2^ vs normal	3.93	1.69	9.14	< 0.01
		Alcohol intake, 1-kcal increase	1.01	1.00	1.01	0.01
Any reason	TPTD-ALN	eGFR, 1-mL/min/1.73 m^2^ increase	1.01	1.00	1.02	0.07
		Calcium, 1-mg/dL increase	0.44	0.28	0.69	< 0.01
	ALN	Age at menopause, 1-year increase	0.95	0.89	1.01	0.09
		MMSE, 1-point increase	0.92	0.87	0.97	< 0.01
		EQ-5D usual activities, 1-point increase	1.43	1.00	2.04	0.05

*OR* odds ratio, *CI* confidence interval, *AE* adverse event, *TPTD *teriparatide, *ALN *alendronate, *P1NP* procollagen type 1 N-terminal propeptide, *BMI *body mass index, *eGFR* estimated glomerular filtration rate, *MMSE* mini-mental state examination

To investigate the factors contributing to serum calcium levels in the TPTD-ALN group, the participants were divided into two groups based on the median serum calcium cut-off value (9.5 mg/dL), and then 25OHVD levels were compared. The 25OHVD level was significantly lower in the lower serum calcium group (16.8 ± 5.8 ng/mL) than in the higher serum calcium group (18.4 ± 5.7 ng/mL; p < 0.01).

## Discussion

In this sub-analysis, the relationships between participants’ baseline clinical parameters and discontinuation were comprehensively investigated. Factors related to discontinuation varied by the reason for discontinuation and treatment regimen. A lower serum calcium level was associated with discontinuations related to compliance and any reason in patients treated with the TPTD-ALN regimen. On the other hand, MMSE was associated with discontinuation related to compliance and any reason in those treated with the ALN monotherapy regimen.

Previously, higher age, incidence of AEs, and lower BMD were reported to be associated with discontinuation of TPTD treatment [19, 20]. The results of the present study suggest that a lower serum calcium level is another possible predictor of discontinuation in patients treated with once-weekly TPTD. Recent reports indicate that non-use of calcium supplements is an independent predictor of self-discontinuation of osteoporosis medication including TPTD [21]. In the present study, serum 25OHVD levels were lower in participants with lower serum calcium levels. Vitamin D deficiency might be involved in lower serum calcium levels. Taken together, evaluation of serum calcium levels before TPTD administration may be useful to predict patients with poor compliance and those who need vitamin D supplementation.

In contrast, a lower MMSE was significantly associated with discontinuation in the ALN group. The MMSE is used to identify mild and severe cognitive impairment with a simple and widely used first-level neurocognitive screening test. Although it has already been reported that mild cognitive impairment (MCI) diagnosed by MMSE is associated with poor adherence to medication [22], there have been few reports regarding cognitive function and treatment discontinuation in patients with osteoporosis. Low MMSE scores can be related to cognitive impairment and involved in poor adherence to ALN prescriptions, because the dosage instructions are more complicated for ALN than for other drugs. In this context, it is interesting to note that the median MMSE in this analysis was 27, which is equal to the MCI cutoff value in Japan.

The prevalence of hyperlipidemia was inversely related to compliance-related discontinuation in the ALN group. Body weight and the circulating osteocalcin level were also associated with compliance-related discontinuation in the ALN group. These observations were not consistent among the reasons for discontinuation, and the causal relationship was unclear.

A higher BMI and alcohol intake were positively associated with AE-related discontinuation in the ALN group. Alcohol and obesity have been reported to be associated with gastrointestinal disorders such as erosive esophagitis and gastroesophageal reflux disease [23]. Gastrointestinal disorders are well-known AEs associated with bisphosphonates including ALN and were observed in 9.4% of the ALN group in the JOINT-05 trial [12]. BMI and alcohol may be related to the incidence of ALN-specific AEs.

Several patient support programs have been tried to continue treatment for osteoporosis, such as a fracture liaison service and pharmacist-delivered counseling. These programs appeared to be successful in improving osteoporosis medication adherence in some settings, whereas behavioral interventions including patient counseling and reminder prompts for medication utilization were not [24]. This was perhaps due to patient perceptions regarding osteoporosis consequences and the need for treatment. Thus, for patients with MCI, their family members should receive exceptionally detailed explanations and understand the consequences of osteoporosis and the need for treatment.

This study had several limitations. The present analysis was based on data from subjects enrolled in a randomized, controlled trial to evaluate the efficacy and safety of an anabolic drug (TPTD). The eligibility criteria stipulated osteoporosis with a higher fracture risk and did not include low-risk groups. In general, patients eligible for bisphosphonate therapy have a broad spectrum of fracture risk. Therefore, another study may be needed to confirm predictors of ALN discontinuation in patients with a lower fracture risk. However, this head-to-head randomized, controlled trial identified predictors of discontinuation specific to each drug for osteoporosis, once-weekly TPTD or ALN, which is a strength of this study. In particular, the parameters associated with discontinuing treatment regimens did not overlap between the TPTD-ALN and ALN groups. This fact suggests that patients with lower serum calcium levels are specifically prone to discontinuation of once-weekly TPTD, whereas low MMSE scores can be specifically predictive of ALN discontinuation. Prospective studies may be needed to test the causal relationships of these factors to discontinuation. In the present study, the timing of dropouts was the date the investigators realized the dropout of participants, but not accurate information regarding the date of dropout that could not be confirmed objectively. Thus, the detailed timing of dropout was not investigated in this study. Therefore, it is difficult to discriminate discontinuation due to ALN from that due to TPTD in the TPTD-ALN group. In addition, the discontinuation rate was higher in the early 72 weeks than in the subsequent 48 weeks [12]. Taken together, the discontinuation in the TPTD-ALN group may still be mainly influenced by TPTD treatment.

In conclusion, predictors of discontinuation were specific to each drug for osteoporosis, once-weekly TPTD or ALN. A lower serum calcium level and poor cognitive function were predictors of discontinuation of treatment with once-weekly TPTD and ALN, respectively. When starting patients on treatment with TPTD and ALN, healthcare providers should pay more careful attention to patients with lower serum calcium levels and poor cognitive function, respectively, for better treatment continuity.

## Supplementary Information

Below is the link to the electronic supplementary material.

Supplementary file1 (DOCX 362 KB)

## Data Availability

The data that support the findings of this study are available from the corresponding author upon reasonable request.
