# Supplementary material for: Predictors of discontinuation of osteoporosis treatment: sub-analysis of the Japanese osteoporosis intervention trial-05 (JOINT-05)
Source: J Bone Miner Metab. 2024 Aug 14;42(6):675–80. doi: 10.1007/s00774-024-01541-3 (PMC11632025; doi:10.1007/s00774-024-01541-3)
Supplement: Supplementary file 1 — Supplementary file1 (DOCX 362 KB) [file 774_2024_1541_MOESM1_ESM.docx]

Asterisks indicate potential predictors identified by the LASSO method.

Supplemental Table 1. Associations between compliance-related treatment discontinuation and participants’ baseline characteristics

Teriparatide (N=478)

Alendronate (N=488)

Total (N=966)

Odds ratio

95% CI

p

Odds ratio

95% CI

p

Odds ratio

95% CI

p

Age (y)

Age at menopause (y) Years from menopause

Number of prevalent vertebral fractures Maximum grade of prevalent vertebral fractures

Grade 1

Grade 2

Grade 3

History of proximal femoral fractures Prior treatment

Prior bisphosphonates BMD (T-score)

BMD at L2-L4 (T-score) Comorbidities

Hypertension Diabetes mellitus Dyslipidemia Rheumatoid arthritis Osteoarthritis Others

MMSE

Timed-up-and-go test

One-leg standing

1.02

0.98

1.02

0.90

0.98

0.94

0.99

0.80

1.07

1.03

1.05

1.01

0.31

0.50

0.24

0.07

1.01

0.97

1.02

0.95

0.97

0.93

0.99

0.85

1.06

1.01

1.05

1.05

0.51

0.18

0.17

0.30

1.02

0.98

1.02

0.92

0.99

0.95

1.00

0.85

1.05

1.01

1.04

1.00

. 1.70

1.38

1.12

1.85

1.03

1.27

1.10

1.13

0.24

0.17

0.08

0.04

. 0.92

0.65

0.21

0.29

0.08

0.68

0.68

0.40

1.19

0.75

0.69

1.08

0.77

1.03

1.01

1.03

0.60

0.41

0.44

0.62

0.52

0.67

0.91

0.92

2.37

1.37

1.10

1.89

1.14

1.57

1.13

1.16

0.62

0.35

0.12

0.78

0.19

0.91

0.81

0.61

0.86

1.10

0.95

1.43

0.78

0.85

1.02

1.04

0.40

0.62

0.59

0.81

0.52

0.55

0.91

0.92

1.85

1.98

1.53

2.52

1.17

1.33

1.13

1.17

0.70

0.74

0.83

0.22

0.23

0.49

0.78

0.53

1.03

0.91

0.81

1.24

0.77

0.94

1.02

1.04

0.62

0.60

0.58

0.83

0.59

0.69

0.94

0.95

0.75

0.88

0.63

0.50

0.43

0.35

1.13

1.81

1.13

0.17

0.73

0.12

0.85

0.83

0.48

0.56

0.40

0.25

1.29

1.74

0.93

0.44

0.62

0.03

0.80

0.85

0.56

0.59

0.51

0.36

1.07

1.43

0.86

0.13

0.55

0.01

Not estimable Not estimable

Not estimable Not estimable

Not estimable Not estimable

0.78

0.98

1.01

1.00

Reference 0.77

1.21

0.41

1.36

2.26

1.03

0.98

1.53

Reference 0.59

1.01

1.00

1.00

1.00

1.00

0.99

1.00

1.01

0.77

1.00

1.01

1.00

1.00

1.01

0.69

1.00

0.58

0.46

1.01

1.00

1.00

0.83

1.31

0.87

0.96

0.90

1.00

1.00

1.01

1.01

1.00

0.94

1.00

1.07

1.00

1.03

1.00

2.72

1.91

1.97

1.08

1.00

1.00

1.00

0.49

0.94

0.99

0.99

1.22

1.03

1.04

1.01

0.27

0.47

0.32

0.96

0.71

0.91

1.00

0.99

Reference 1.05

0.83

2.04

5.82

1.46

0.98

0.97

1.38

Reference 0.65

1.00

1.00

1.02

1.00

1.00

0.98

1.00

1.00

0.90

1.00

1.00

1.00

1.00

1.01

0.78

1.00

0.77

0.71

1.01

1.00

1.00

1.13

1.25

1.28

1.27

1.23

0.99

1.00

1.00

1.00

1.00

0.98

1.00

1.03

1.00

1.00

1.00

1.44

1.22

1.51

1.02

1.00

1.00

1.00

0.45

0.87

0.99

0.98

1.14

0.96

1.02

1.00

0.16

<0.01

0.65

0.21

0.74

0.95

1.01

1.00

Reference 0.90

1.06

1.10

2.14

1.54

1.01

0.97

1.48

Reference 0.62

1.00

1.00

1.01

1.00

1.00

0.99

1.00

1.01

0.85

1.00

1.01

1.00

1.00

1.01

0.73

1.00

0.66

0.56

1.01

1.00

1.00

0.96

1.29

1.05

1.10

1.06

1.00

1.00

1.01

1.01

1.00

0.96

1.00

1.05

1.00

1.02

1.00

2.02

1.51

1.75

1.05

1.00

1.00

1.00

0.54

0.92

0.99

0.99

1.03

0.98

1.02

1.00

Nursing level

Support not required Support required Level 1

Level 2

Level 3

Level 4 or 5

0.41

0.50

0.09

0.32

0.14

1.00

0.96

0.92

1.44

2.93

1.89

5.78

36.50

1.06

1.00

2.56

0.42

0.68

0.25

0.68

0.56

0.07

0.05

0.10

0.54

0.27

0.76

0.52

0.26

0.95

0.94

0.79

2.06

2.58

5.49

64.89

8.07

1.01

0.99

2.42

0.88

0.75

0.16

0.15

0.67

0.26

0.01

0.26

0.57

0.53

0.50

0.65

0.36

0.99

0.96

1.01

1.42

2.12

2.44

7.08

6.50

1.03

0.99

2.16

0.65

0.86

0.81

0.21

0.56

0.53

<0.01

0.04

Height Weight BMI BMI

BMI

<18.5 kg/m2

18.5 to 24 kg/m2

≥25 kg/m2

0.35

1.00

0.99

0.98

1.00

1.00

0.96

1.00

1.00

0.52

0.99

1.00

0.99

0.99

1.00

0.28

1.00

0.34

0.28

0.99

1.00

0.99

0.57

0.88

0.60

0.69

0.60

0.99

1.00

1.00

1.00

0.99

0.84

1.00

0.99

1.00

0.96

1.00

1.16

1.21

1.03

1.03

1.00

1.00

1.00

1.00

1.01

1.02

1.01

1.01

1.00

1.03

1.01

1.01

1.14

1.00

1.02

1.00

1.00

1.02

1.71

1.01

0.99

0.74

1.03

1.01

1.01

1.20

1.95

1.24

1.35

1.35

1.00

1.00

1.02

1.02

1.00

1.04

1.00

1.16

1.00

1.11

1.00

6.35

3.01

3.78

1.13

1.00

1.01

1.01

0.05

0.13

0.78

0.70

0.03

0.19

0.61

0.22

0.16

0.19

0.90

0.03

0.29

0.33

0.03

0.43

0.18

0.05

<0.01

0.48

0.40

0.96

0.32

0.18

0.44

0.82

0.61

0.63

0.49*

0.06

0.13

0.53

0.23

0.11

0.07

0.02*

0.40

0.07

0.02

0.01

0.04

<0.01

0.01

0.54

0.12

0.37

0.99

0.99

1.00

1.00

1.00

0.95

1.00

0.99

0.65

0.99

0.99

0.99

0.99

1.00

0.28

1.00

0.43

0.42

0.99

0.99

1.00

0.76

0.81

0.89

0.90

0.85

0.99

1.00

0.99

0.99

1.00

0.88

1.00

0.95

1.00

0.93

1.00

0.59

0.80

0.76

0.97

1.00

1.00

1.00

1.15

1.01

1.02

1.04

1.01

1.00

1.02

1.01

1.02

1.23

1.00

1.01

1.00

1.00

1.02

2.16

1.01

1.36

1.21

1.04

1.01

1.01

1.68

1.93

1.85

1.80

1.80

1.00

1.00

1.01

1.02

1.01

1.09

1.00

1.12

1.00

1.08

1.00

3.46

1.88

3.01

1.07

1.00

1.01

1.00

0.14

0.60

0.49

0.02

0.17

0.19

0.36

0.36

0.36

0.50

0.30

0.81

0.57

0.05

0.17

0.63

0.37

0.37

0.21

0.42

0.99

0.63

0.53

0.31

0.19

0.17

0.27

0.17

0.61

0.40

0.54

0.71

0.72

0.93

0.44

0.22

0.96

0.51

0.42

0.36

0.24

0.52

0.34

0.27

0.64

0.42

1.00

0.99

1.00

1.00

1.00

0.96

1.00

1.00

0.66

0.99

1.00

0.99

0.99

1.00

0.37

1.00

0.45

0.39

0.99

1.00

1.00

0.73

0.96

0.81

0.86

0.80

0.99

1.00

1.00

1.00

1.00

0.89

1.00

1.00

1.00

0.96

1.00

1.10

1.11

1.09

1.01

1.00

1.00

1.00

0.92

1.01

1.01

1.02

1.01

1.00

1.01

1.01

1.01

1.09

1.00

1.02

1.00

1.00

1.02

1.44

1.00

0.97

0.79

1.03

1.01

1.01

1.26

1.73

1.36

1.40

1.40

1.00

1.00

1.01

1.02

1.00

1.03

1.00

1.11

1.00

1.07

1.00

3.72

2.05

2.81

1.08

1.00

1.01

1.00

0.02

0.48

0.49

0.17

0.01

0.07*

0.33

0.12

0.08

0.19

0.46

0.07

0.26

0.03

0.01

0.36

0.10

0.04

<0.01

0.28

0.64

0.71

0.77

0.09

0.71

0.44

0.67

0.18

0.37*

0.04

0.12

0.86

0.25

0.23*

0.07

0.01*

0.54

0.09

0.02

0.01

0.02

0.01

0.01

0.24

0.15

Systolic blood pressure Diastolic blood pressure Osteocalcin

P1NP TRACP-5b 25OHVD

Pentosidine

Corrected pentosidine HbA1C

Total cholesterol HDL cholesterol LDL cholesterol Triglycerides

eGFR

Creatinine

Urine creatinine Albumin Calcium

Urine calcium

VAS at rest VAS on motion EQ-5D (mobility)

EQ-5D (self-care)

EQ-5D (usual activities) EQ-5D (pain/discomfort)

EQ-5D (anxiety/depression) Alcohol intake

Energy intake Protein intake Fat intake

Carbohydrate intake Salt intake

Ca intake Fe intake

Vitamin A intake Vitamin D intake Vitamin K intake Vitamin B1 intake Vitamin B2 intake Vitamin B6 intake Vitamin B12 intake Folic acid intake Vitamin C intake Mg intake

0.07

<0.01

0.37

0.37

Supplemental Table 2. Associations between adverse-event-related treatment discontinuation and participants’ baseline characteristics

Asterisks indicate potential predictors identified by the LASSO method.

0.04

0.21

0.60

0.93

0.68

0.97

0.11

0.33

0.62

0.09

0.52

0.20

0.83

0.66

0.75

0.57

0.40

0.75

0.08

0.07

0.90

0.85

0.73

0.88

0.38

0.96

0.26

0.39

0.13

0.31

0.26

0.10

0.77

0.95

0.74

0.26*

0.24

0.66

0.20

0.39

0.37

0.18

0.31

0.75

0.51

0.89

0.29

0.95

0.78

0.10

0.38

0.17

0.54

0.61

0.58

0.58

0.27

0.85

0.66

0.29

0.55

0.44

0.64

0.13

0.33

0.83

0.76

0.73

0.45

0.72

0.99

0.95

0.53

0.35

0.83

0.88

0.10

0.83

0.43

0.86

0.42*

0.04

0.56

0.48

0.62

0.83

0.28

0.46

0.79

0.76

Teriparatide (N=478)

Alendronate (N=488)

Total (N=966)

Odds ratio

95% CI

p

Odds ratio

95% CI

p

Odds ratio

95% CI

p

0.97

1.05

0.96

0.93

0.92

0.99

0.93

0.81

1.03

1.11

1.00

1.07

0.29

0.11

0.07

0.32

1.07

0.90

1.09

1.03

1.00

0.85

1.04

0.87

1.15

0.96

1.14

1.22

0.07

<0.01

<0.01

0.70

1.00

0.99

1.00

0.96

0.96

0.95

0.97

0.87

1.05

1.04

1.04

1.07

0.87

0.81

0.78

0.50

0.31

0.81

0.87

0.77

1.15

1.14

1.00

0.99

0.09

0.39

0.50

0.36

0.70

0.68

0.87

0.85

1.06

1.69

1.50

1.63

1.87

1.92

1.15

1.14

0.06

0.57

0.61

0.49

0.58

0.62

0.99

0.87

2.79

0.67

1.64

0.91

0.69

0.60

0.93

0.92

0.92

0.17

0.68

0.31

0.34

0.25

0.77

0.74

8.52

2.58

3.93

2.67

1.40

1.41

1.13

1.13

0.07

0.55

0.27

0.86

0.30

0.24

0.49

0.42

0.81

0.75

1.05

0.83

0.97

0.94

0.99

0.97

0.38

0.40

0.67

0.45

0.65

0.61

0.89

0.87

1.75

1.41

1.66

1.53

1.44

1.45

1.10

1.10

0.60

0.37

0.82

0.55

0.88

0.77

0.84

0.67

1.22

1.81

1.19

0.75

0.85

0.63

2.00

3.88

2.26

0.42

0.13

0.59

1.24

1.47

1.53

9.72

0.60

0.49

0.64

1.57

2.53

4.40

3.67

60.34

0.56

0.49

0.34

0.01

1.23

1.64

1.30

3.12

0.82

0.89

0.78

0.60

1.83

3.03

2.17

16.27

0.32

0.11

0.31

0.18

Not estimable Not estimable

Not estimable

Not estimable

1.23

1.01

1.00

1.00

Reference 1.30

0.49

1.53

0.73

0.95

0.97

0.99

2.09

1.07

1.03

1.01

0.44

0.70

0.98

0.86

0.96

0.95

1.02

0.99

Reference 0.89

1.70

0.85

0.43

0.88

1.00

0.98

2.12

1.02

1.04

1.01

0.92

0.16

0.03

0.58

1.12

0.99

1.01

1.00

Reference 1.24

0.83

1.19

0.73

0.94

0.99

0.99

1.73

1.03

1.03

1.01

0.60

0.54

0.19

0.87

Nursing level

Support not required Support required Level 1

Level 2

Level 3

Level 4 or 5

0.65

0.11

0.41

2.60

2.12

5.72

0.45

0.34

0.53

0.26

0.37

0.11

3.05

7.80

6.67

0.85

0.49

0.88

0.69

0.29

0.40

2.24

2.40

3.48

0.47

0.74

0.75

Not estimable Not estimable

Not estimable Not estimable

Not estimable Not estimable

Height Weight BMI BMI

BMI

1.00

1.00

0.99

Reference 0.96

0.99

1.00

1.00

0.99

1.00

1.03

1.00

1.00

1.13

1.00

0.99

1.00

1.00

1.01

0.70

1.00

1.17

0.64

0.99

1.00

1.00

1.08

0.81

0.92

1.00

1.01

1.00

1.00

1.00

1.00

1.00

1.01

1.00

0.99

1.00

1.10

1.00

0.69

0.86

0.92

0.97

1.00

1.00

1.00

0.97

0.98

0.51

1.04

1.03

1.93

0.88

0.78

0.97

0.96

1.04

1.30

Reference 3.67

1.00

0.99

1.01

1.00

1.00

1.02

1.00

1.01

1.37

1.00

0.98

1.00

1.00

0.97

3.66

1.00

0.56

0.69

0.95

1.00

1.00

1.01

1.30

1.84

1.11

1.79

1.01

1.00

0.98

0.97

1.00

0.95

1.00

0.98

1.00

0.96

1.00

0.31

0.71

0.40

0.97

1.00

0.99

1.00

0.91

1.00

0.42

1.01

1.08

4.03

0.14

0.05

0.65

0.99

1.02

1.15

Reference 1.62

0.99

1.00

1.00

1.00

1.00

1.03

1.00

1.00

1.25

1.00

0.99

1.00

1.00

1.00

1.29

1.00

0.91

0.64

0.98

1.00

1.00

1.07

0.97

1.17

1.01

1.24

1.00

1.00

0.99

0.99

1.00

0.98

1.00

0.99

1.00

1.05

1.00

0.58

0.82

0.74

0.97

1.00

1.00

1.00

0.96

0.99

0.65

1.02

1.04

2.02

0.63

0.16

0.64

<18.5 kg/m2

18.5 to 24 kg/m2

≥25 kg/m2

0.52

0.98

0.98

0.97

0.99

1.00

0.99

0.99

0.99

0.73

0.99

0.98

0.99

1.00

0.99

0.23

1.00

0.59

0.36

0.96

0.99

0.99

0.69

0.48

0.59

0.66

0.62

0.99

1.00

0.99

0.99

0.99

0.89

1.00

0.90

1.00

1.00

1.00

0.25

0.49

0.41

0.91

1.00

0.99

0.99

1.75

1.00

1.02

1.02

1.00

1.00

1.07

1.01

1.01

1.76

1.00

1.01

1.01

1.00

1.02

2.20

1.01

2.32

1.15

1.01

1.01

1.01

1.71

1.39

1.44

1.52

1.67

1.01

1.00

1.01

1.02

1.00

1.15

1.00

1.09

1.00

1.21

1.00

1.93

1.53

2.03

1.03

1.00

1.01

1.00

1.70

0.98

0.97

0.97

1.00

1.00

0.96

0.99

1.00

0.98

0.99

0.96

0.99

1.00

0.95

0.99

0.99

0.21

0.27

0.89

0.99

0.99

0.50

0.63

0.99

0.61

0.99

1.00

1.00

0.96

0.95

0.99

0.78

1.00

0.85

1.00

0.84

1.00

0.07

0.31

0.12

0.88

1.00

0.98

0.99

7.93

1.01

1.01

1.04

1.01

1.00

1.09

1.02

1.03

1.92

1.01

1.01

1.01

1.01

0.99

13.56

1.01

1.51

1.75

1.00

1.02

1.02

2.03

2.70

3.42

2.02

3.23

1.01

1.00

1.00

0.99

1.01

1.15

1.00

1.13

1.00

1.09

1.00

1.45

1.61

1.30

1.06

1.00

1.01

1.00

<0.01

0.56

0.35

0.75

0.15

0.17*

0.47

0.56

0.10

0.07

0.45

0.18

0.68

0.66

0.02

0.05

0.98

0.25

0.43

0.06

0.55

0.42

0.97

0.48

0.05

0.74

0.05

0.04

0.11*

0.04

0.01

0.61

0.59

0.26

0.75

0.59*

0.49

0.92

0.14

0.41

0.13

0.50

0.63

0.37

0.46

1.02

0.99

0.98

0.98

0.99

1.00

0.99

1.00

0.99

0.96

0.99

0.98

0.99

1.00

0.99

0.54

1.00

0.53

0.39

0.95

0.99

0.99

0.73

0.63

0.82

0.72

0.85

1.00

1.00

0.99

0.98

0.99

0.89

1.00

0.91

1.00

0.97

1.00

0.25

0.52

0.38

0.92

1.00

0.99

0.99

2.57

1.00

1.01

1.02

1.00

1.00

1.06

1.01

1.01

1.63

1.00

1.00

1.01

1.00

1.01

3.08

1.00

1.59

1.05

1.00

1.01

1.01

1.56

1.48

1.68

1.42

1.81

1.01

1.00

1.00

1.01

1.00

1.09

1.00

1.07

1.00

1.13

1.00

1.35

1.29

1.43

1.02

1.00

1.01

1.00

Systolic blood pressure Diastolic blood pressure Osteocalcin

P1NP TRACP-5b 25OHVD

Pentosidine

Corrected pentosidine HbA1C

Total cholesterol HDL cholesterol LDL cholesterol Triglycerides

eGFR

Creatinine

Urine creatinine Albumin Calcium

Urine calcium VAS at rest VAS on motion EQ-5D (mobility)

EQ-5D (self-care)

EQ-5D (usual activities) EQ-5D (pain/discomfort)

EQ-5D (anxiety/depression) Alcohol intake

Energy intake Protein intake Fat intake

Carbohydrate intake Salt intake

Ca intake Fe intake

Vitamin A intake Vitamin D intake Vitamin K intake Vitamin B1 intake Vitamin B2 intake Vitamin B6 intake Vitamin B12 intake Folic acid intake Vitamin C intake Mg intake

Age (y)

Age at menopause (y) Years from menopause

Number of prevalent vertebral fractures Maximum grade of prevalent vertebral fractures

Grade 1

Grade 2

Grade 3

History of proximal femoral fractures Prior treatment

Prior bisphosphonates BMD (T-score)

BMD at L2-L4 (T-score) Comorbidities

Hypertension Diabetes mellitus Dyslipidemia Rheumatoid arthritis Osteoarthritis Others

MMSE

Timed-up-and-go test

One-leg standing

Asterisks indicate potential predictors identified by the LASSO method.

Supplemental Table 3. Associations between all-cause treatment discontinuation and participants’ baseline characteristics

0.63

0.31

0.99*

0.01*

0.05*

0.06*

0.59

0.22

0.09

0.56

0.16

0.50

0.52

0.10*

0.69

0.29

0.40*

0.06

0.07

0.52

0.56

0.31

0.29

0.14

0.01

0.18

0.08

0.80*

0.75*

0.68

0.38

0.76*

0.66

0.59*

0.61

0.48*

0.96

0.82*

0.85

0.85

0.92

0.97

0.68

0.52

0.90

0.83

0.62

0.24

0.26

0.99

0.09*

0.23

0.28

0.15

<0.01

0.15

0.26

0.14

<0.01

0.11*

0.09*

0.11

<0.01

<0.01

0.43

Teriparatide (N=478)

Alendronate (N=488)

Total (N=966)

Odds ratio

95% CI

p

Odds ratio

95% CI

p

Odds ratio

95% CI

p

Age (y)

Age at menopause (y) Years from menopause

Number of prevalent vertebral fractures Maximum grade of prevalent vertebral fractures

Grade 1

Grade 2

Grade 3

History of proximal femoral fractures Prior treatment

Prior bisphosphonates BMD (T-score)

BMD at L2-L4 (T-score) Comorbidities

Hypertension Diabetes mellitus Dyslipidemia Rheumatoid arthritis Osteoarthritis Others

MMSE

Timed-up-and-go test

One-leg standing

1.01

1.02

1.00

0.92

0.97

0.98

0.97

0.83

1.05

1.06

1.03

1.01

0.52

0.44

0.94

0.08

1.03

0.94

1.05

0.96

0.99

0.90

1.02

0.88

1.07

0.98

1.08

1.06

1.02

0.98

1.02

0.94

0.99

0.95

1.00

0.88

1.05

1.01

1.04

1.00

. 1.48

1.19

1.20

1.65

1.00

1.14

1.10

1.12

. 1.16

1.62

1.01

2.91

0.13

0.19

0.05

0.06

. 0.74

0.29

0.45

0.49

0.05

0.29

0.50

0.35

. 0.39

0.90

0.06

0.49

0.73

0.71

0.72

0.95

0.86

1.02

1.02

1.03

0.37

0.41

0.47

0.56

0.60

0.69

0.92

0.92

1.42

1.23

1.09

1.59

1.24

1.51

1.13

1.15

0.35

0.23

0.12

0.83

0.43

0.92

0.68

0.59

1.18

0.96

1.10

1.37

0.68

0.71

1.02

1.03

0.60

0.55

0.71

0.80

0.47

0.47

0.92

0.92

2.35

1.68

1.72

2.35

1.00

1.08

1.12

1.16

0.63

0.88

0.66

0.25

0.05

0.11

0.77

0.57

0.92

0.81

0.89

1.14

0.78

0.86

1.02

1.04

0.57

0.55

0.66

0.79

0.60

0.65

0.95

0.96

0.91

1.17

0.72

0.00

0.63

0.61

0.44

0.00

1.32

2.23

1.18

0.63

0.64

0.19

1.00

0.86

0.92

0.66

1.28

0.58

0.47

0.38

0.21

1.26

1.80

1.14

7.77

0.43

0.81

0.14

0.78

0.89

1.03

0.70

0.56

0.68

0.65

0.49

0.11

Not estimable

Not estimable

Not estimable

0.89

0.98

1.01

1.00

Reference 0.98

0.78

0.87

0.61

1.01

1.02

0.98

1.37

Reference 0.64

1.00

1.00

1.00

1.00

1.00

1.01

1.00

1.00

0.82

1.00

1.00

1.00

1.00

1.01

0.67

1.00

0.69

0.41

1.00

1.01

1.00

1.01

1.16

0.92

0.99

0.92

1.00

1.00

1.01

1.01

1.00

0.93

1.00

1.05

1.00

1.08

1.00

1.64

1.51

1.62

1.04

1.00

1.00

1.00

0.60

0.94

0.98

0.99

1.34

1.03

1.03

1.01

0.59

0.42

0.57

0.99

0.73

0.91

1.01

0.99

Reference 1.07

1.28

1.79

4.03

1.01

0.97

0.99

1.35

Reference 1.13

1.00

1.00

1.02

1.00

1.00

0.99

1.00

1.01

1.08

1.00

1.00

1.00

1.00

1.00

1.63

1.00

0.60

0.64

0.99

1.00

1.00

1.22

1.36

1.56

1.25

1.37

1.00

1.00

1.00

0.99

1.00

0.98

1.00

1.02

1.00

1.00

1.00

0.92

1.04

1.03

1.00

1.00

1.00

1.00

0.47

0.86

1.00

0.98

1.12

0.95

1.03

1.00

0.81

0.95

1.01

1.00

Reference 1.06

1.01

1.25

1.19

0.86

1.00

0.98

1.41

Reference 0.87

1.00

1.00

1.01

1.00

1.00

1.00

1.00

1.01

0.98

1.00

1.00

1.00

1.00

1.01

0.99

1.00

0.65

0.50

1.00

1.00

1.00

1.10

1.26

1.19

1.09

1.13

1.00

1.00

1.00

1.00

1.00

0.95

1.00

1.04

1.00

1.03

1.00

1.30

1.24

1.34

1.02

1.00

1.00

1.00

0.61

0.92

1.00

0.99

1.08

0.98

1.02

1.00

Nursing level

Support not required Support required Level 1

Level 2

Level 3

Level 4 or 5

0.56

0.33

0.29

0.14

0.06

0.99

0.96

0.83

1.69

1.82

2.63

2.57

16.28

1.05

1.00

2.26

0.93

0.56

0.80

0.50

0.99

0.13

0.08

0.23

0.57

0.49

0.68

0.36

0.18

0.95

0.96

0.79

2.00

3.38

4.75

44.86

5.57

1.00

1.01

2.31

0.70

0.53

0.60

0.36

0.20

0.98

0.97

0.98

1.59

1.91

2.60

3.93

3.61

1.02

1.00

2.03

0.78

0.97

0.55

0.78

0.83

0.87

0.05

0.06

Height Weight BMI BMI

BMI

<18.5 kg/m2

18.5 to 24 kg/m2

≥25 kg/m2

0.41

0.99

0.99

0.98

1.00

1.00

0.98

1.00

1.00

0.58

0.99

0.99

0.99

1.00

1.00

0.30

1.00

0.42

0.26

0.98

1.00

0.99

0.72

0.80

0.66

0.73

0.64

0.99

1.00

1.00

0.99

0.99

0.84

1.00

0.98

1.00

1.00

1.00

0.76

0.99

0.89

1.00

1.00

1.00

1.00

1.01

1.01

1.02

1.01

1.01

1.00

1.04

1.01

1.01

1.16

1.00

1.02

1.00

1.00

1.02

1.48

1.01

1.14

0.64

1.02

1.01

1.01

1.42

1.69

1.27

1.35

1.34

1.00

1.00

1.01

1.02

1.00

1.03

1.00

1.13

1.00

1.16

1.00

3.53

2.30

2.93

1.09

1.00

1.01

1.01

0.06

0.70

0.69

0.57

0.34

0.56

0.60

0.14

0.40

0.26

0.66

0.41

0.32

0.99

0.02

0.32

0.07

0.15

<0.01

0.84

0.18

0.87

0.94

0.44

0.60

0.95

0.67

0.38

0.64*

0.25

0.34

0.05

0.15

0.11

0.15

0.10*

0.04

0.03

0.21

0.06

0.11

0.08

0.07

0.51

0.28

0.69

0.99

0.99

1.00

1.00

1.00

0.96

1.00

1.00

0.83

0.99

0.98

0.99

0.99

0.99

0.66

1.00

0.35

0.39

0.97

1.00

1.00

0.84

0.91

1.11

0.91

0.97

0.99

1.00

0.99

0.98

1.00

0.89

1.00

0.95

1.00

0.93

1.00

0.41

0.69

0.55

0.95

1.00

1.00

1.00

1.83

1.00

1.01

1.04

1.01

1.00

1.02

1.01

1.02

1.40

1.00

1.01

1.00

1.00

1.01

3.99

1.01

1.03

1.04

1.02

1.01

1.01

1.76

2.03

2.20

1.72

1.95

1.00

1.00

1.01

1.01

1.00

1.08

1.00

1.10

1.00

1.07

1.00

2.09

1.56

1.95

1.05

1.00

1.01

1.00

0.62

0.99

0.99

1.00

1.00

1.00

0.98

1.00

1.00

0.80

0.99

0.99

0.99

1.00

1.00

0.55

1.00

0.45

0.36

0.98

1.00

1.00

0.86

0.96

0.94

0.87

0.87

0.99

1.00

1.00

0.99

1.00

0.89

1.00

0.98

1.00

0.99

1.00

0.75

0.93

0.87

0.99

1.00

1.00

1.00

1.20

1.00

1.01

1.02

1.01

1.00

1.02

1.01

1.01

1.21

1.00

1.01

1.00

1.00

1.01

1.79

1.00

0.93

0.69

1.01

1.01

1.01

1.41

1.65

1.50

1.36

1.45

1.00

1.00

1.01

1.01

1.00

1.02

1.00

1.09

1.00

1.09

1.00

2.25

1.65

2.06

1.05

1.00

1.01

1.00

0.39

0.63

0.81

0.20

0.03

0.11

0.92

0.04

0.07

0.87

0.30

0.63

0.32

0.18

0.11

0.98

0.04

0.02

<0.01

0.62

0.36

0.42

0.43

0.10

0.15

0.45

0.36

0.46

0.69*

0.39

0.78

0.24

0.14

0.41

0.18

0.16*

0.17

0.12

0.35

0.15

0.18

0.24

0.17

0.39

0.50

Systolic blood pressure Diastolic blood pressure Osteocalcin

P1NP TRACP-5b 25OHVD

Pentosidine

Corrected pentosidine HbA1C

Total cholesterol HDL cholesterol LDL cholesterol Triglycerides

eGFR

Creatinine

Urine creatinine Albumin Calcium

Urine calcium VAS at rest VAS on motion EQ-5D (mobility)

EQ-5D (self-care)

EQ-5D (usual activities) EQ-5D (pain/discomfort)

EQ-5D (anxiety/depression) Alcohol intake

Energy intake Protein intake Fat intake

Carbohydrate intake Salt intake

Ca intake Fe intake

Vitamin A intake Vitamin D intake Vitamin K intake Vitamin B1 intake Vitamin B2 intake Vitamin B6 intake Vitamin B12 intake Folic acid intake Vitamin C intake Mg intake
